# RPA using a multiplexed cartridge for low cost point of care diagnostics in the field

**DOI:** 10.1016/j.ab.2018.02.010

**Published:** 2018-04-15

**Authors:** Luck Tosan Ereku, Ruth E. Mackay, Pascal Craw, Angel Naveenathayalan, Thomas Stead, Manorharanehru Branavan, Wamadeva Balachandran

**Affiliations:** a Centre for Electronic Systems Research, Electronic and Computer Engineering, CEDPS, Brunel University London, Kingston Lane, Uxbridge UB8 3PH, UK; b Mechanical, Aerospace and Civil Engineering, CEDPS, Brunel University London, Kingston Lane, Uxbridge UB8 3PH, UK; c Oceans and Atmosphere Flagship, Commonwealth Science and Industrial Research Organisation (CSIRO), Hobart, Tasmania 7001, Australia

**Keywords:** RPA, Point of care, DNA extraction, Isothermal amplification, Multiplexed cartridge

## Abstract

A point of care device utilising Lab-on-a-Chip technologies that is applicable for biological pathogens was designed, fabricated and tested showing sample in to answer out capabilities. The purpose of the design was to develop a cartridge with the capability to perform nucleic acid extraction and purification from a sample using a chitosan membrane at an acidic pH. Waste was stored within the cartridge with the use of sodium polyacrylate to solidify or gelate the sample in a single chamber. Nucleic acid elution was conducted using the RPA amplification reagents (alkaline pH). Passive valves were used to regulate the fluid flow and a multiplexer was designed to distribute the fluid into six microchambers for amplification reactions. Cartridges were produced using soft lithography of silicone from 3D printed moulds, bonded to glass substrates. The isothermal technique, RPA is employed for amplification. This paper shows the results from two separate experiments: the first using the RPA control nucleic acid, the second showing successful amplification from *Chlamydia Trachomatis*.

Endpoint analysis conducted for the RPA analysis was gel electrophoresis that showed 143 base pair DNA was amplified successfully for positive samples whilst negative samples did not show amplification. End point analysis for *Chlamydia Trachomatis* samples was fluorescence detection that showed successful detection of 1 copy/μL and 10 copies/μL spiked in a MES buffer.

## Introduction

Point of care (POC) diagnostics for molecular diagnostics have been the focus of many research projects since the beginning of the century. Whilst the GeneXpert has seen success, the four module platform has never been seen as truly point of care. This has changed with the advent of the GeneXpert Omni – a portable battery powered POC diagnostic; however the limitation to run a single diagnostic in one run is a limitation and benchtop sample preparation is still required [1]. POC devices such as the GeneXpert use nucleic acid (NA) amplification for the detection of pathogens. This paper focuses on the amplification of double stranded nucleic acid, deoxyribonucleic acid (DNA) found in bacteria. The GeneXpert uses the most common method for NA detection, polymerase chain reaction (PCR) [2]. PCR requires thermal cycling through three reaction temperatures 95, 50 and 72 ^°^C. Two primers are required for PCR, nucleic acid is doubled in each thermal cycle, overall the reaction is slower than isothermal equivalents and the requirement for thermal cycling has made it a less popular solution at the point of care due to the high power requirements [3,4]. Fluidic devices have been developed incorporating PCR using fluorescence markers, capillary gel electrophoresis and DNA microarrays with volumes ranging from 4 to 50 μL [4].

Isothermal techniques amplify nucleic acids at a single temperature and have been shown as an alternative to PCR. The techniques can be integrated with gel electrophoresis, optical detection and more recently nucleic acid over lateral flow (NALF) [[5], [6], [7]]. The low power consumption required allows the devices to be run using batteries making them portable, low cost and of use in the developing world. Loop mediated amplification (LAMP) uses a set of four primers, a forward inner primer, a backward inner primer and two outer primers. It produces an exponential amplification of the target sequence and produces a comparable number of copies (10^9^) of DNA to PCR within 1 h at 60–65 °C. LAMP can be integrated with colorimetric dyes for ease of detection. Helicase dependent amplification (HDA) utilizes the helicase enzyme to replicate DNA. The method operates at 60–65 °C and incorporates template separation through unwinding of dsDNA, this is followed by the forward and reverse primers binding and polymerase elongation; the process then repeats asynchronously. The process takes 60 min for 10^6^ fold amplification. Assay design is comparatively simple, however the process is slow and amplification has shown relatively low copy numbers. NASBA is a method used in RNA detection, it is therefore of greater use in viral diagnostics; amplification forms cDNA from an RNA template. The process takes 90 min to produce 10^9^ copies of DNA for detection and the operating temperature is low, 41 °C, however, to reduce non-specific amplification a prior step of 65 °C is required. Rolling circle amplification (RCA) is a low temperature, 37 °C, enzymatic method that uses strand displacement through the polymerase activity of the Phi29 bacteriophage DNA polymerase. RCA shows amplification of 10^3^ in 1 h and can be detected using fluorophores or gel electrophoresis [5,8].

Recombinase Polymerase Amplification (RPA) is an isothermal process that utilizes two primers, the reaction runs at temperatures between 37 and 42 °C. RPA can be run rapidly with results being shown in 10 min with complete runs for low copy numbers taking around 30 min to complete. The number of copies produced is 10^9^-10^11^ which is comparable, if not higher than conventional PCR. RPA can be integrated with NALF and gel electrophoresis, optical detection is conducted using a fluorophore. The main advantages of RPA is its simplicity, using only two primers simplifying, primer design, rapid detection time, low operating temperature and the different detection techniques that can be utilized making this a versatile assay for use at the point of care [[9], [10], [11], [12]].

Within the developing world there is a need for new diagnostics at the point of care that are low cost, do not require mains power and are modular. The device presented in this paper is a battery operated, handheld, molecular diagnostic platform that has shown isothermal techniques including HDA [3] and RPA. The handheld device is low cost being fabricated using 3D printed enclosure and off the shelf electronic components, an Arduino Mega is used for electronic control. Three custom boards and PCB spacers make up the main structural isothermal platform. The design, manufacture and tests of a fluidic are shown using the RPA positive reaction kit and then using *Chlamydia Trachomatis* (CT) cells spiked into an MES buffer solution.

## Materials and methods

### Fluidic cartridge design and fabrication

**Fig. 1:**
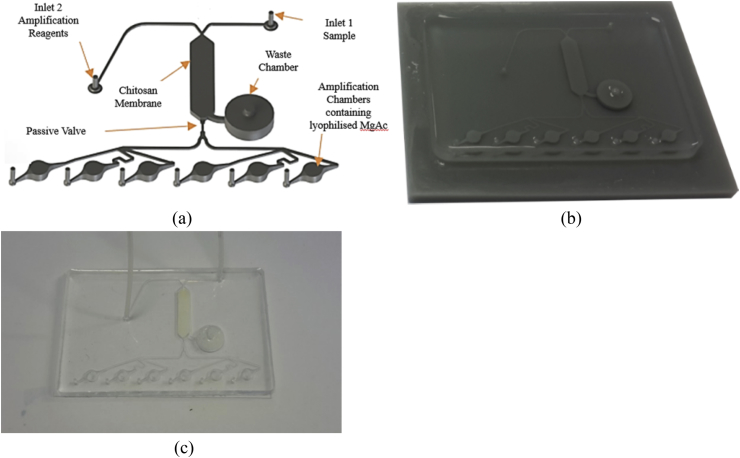
Overview of the fluidic device; (a) Fluidic components showing specific areas for sample input, nucleic acid purification (chitosan membrane), waste storage and amplification/detection; (b) a cured PDMS device attached to 3D printed positive mould; (c) a fabricated device prior to testing incorporating the chitosan membrane and sodium polyacrylate.

The fluidic cartridges of 75 × 50 mm, were designed using the Ansys multiphase CFD simulation software. In the fluidic cartridge (Fig. 1a), there are three reaction chambers. The first is the central reaction chamber that has a volume of 0.65 μL and houses the chitosan membrane, which has a thickness of 100 μm. The chitosan membrane was utilized for DNA extraction on the cartridge. Chitosan is a polysaccharide that can be used for nucleic acid extraction. It has been shown to adsorb DNA in microfluidic devices as it becomes cationic when in acidic conditions due to the protonation of amino groups; when in alkaline conditions DNA is released as protonation no longer occurs neutralising the membrane [13,14]. This reduces the complexity of sample preparation. The second reaction chamber was the waste chamber where bio-waste due to DNA purification in the central chamber was flushed and gelified with sodium polyacrylate, a superabsorbent powder. This waste reservoir has a capacity of 200 μL. The final reaction chambers were designated for the amplification reaction and each have a total volume of 25 μL.

A passive valve was developed and implemented for precise flow regulation, contamination reduction and to provide satisfactory DNA yield. This was expected to redirect unwanted fluid towards the waste chamber for treatment and then, once full, direct fluid towards the amplification chambers. The purpose of the passive device was critical to avoid complicated fluid control devices such as active fluid valves. The functionality of the passive valve design is dependent on three key factors. The first two were geometrically constrained features that involve the use of a narrower channel to bridge to similar channels of equal geometries. Whilst the other features were the accumulation of dead volume as fluids transition from a large domain to a smaller region. The third factor is the surface properties of a hydrophobic substrate that provide sufficient resistance due to unregulated flow by capillarity. As a result, pressure variation becomes the only means to propel and control flow. The general fluidic channel dimension was 1 × 0.5 mm whilst the passive valve had a dimension of 0.3 × 0.5 mm. The final modular element was the multiplexer into which DNA must flow from the passive valves. The multiplexer, designed using Ansys CFD software was tested and allowed accurate filling of all six chambers. The amplification chambers were designed empirically to avoid bubble formation [3]. All the modular elements inclusive of the chamber and the micro channels were fabricated with a standard replication moulding techniques, soft lithography from 3D printed moulds [3]. The use of 3D printing allowed rapid turnaround time of moulds.

The positive master mould has dimensions 70 × 90 mm and was drawn using the CAD package Solidworks 2015 (Dassault Systems) and manufactured using 3D printing on a Viper SLA (3D Systems, USA). Dow Corning's Sylgard 184 was used to create the fluidic channels and chambers. This two-part chemical system involves a mix ratio of cross-linking agent A with siloxane agent B. The mix ratio was 1:10 (Part A: Part B). Upon mixing, the (poly)dimethylsiloxane (PDMS) solution was rapidly stirred by hand for 1–2 min and then placed in a vacuum degassing chamber where the air is removed using an Edward's vacuum pump at regular intervals for 15 min. The degassed PDMS was poured into the positive 3D printed mould and left to cure at 55 °C for 4 h. The cured PDMS was removed from the mould (Fig. 1b); a chitosan membrane was installed in the central reaction chamber, 10 mg of sodium polyacrylate was loaded into the waste chamber and 2.5  μl of magnesium acetate was loaded into each of the amplification reaction chambers. Afterwards, a handheld corona treater, BD20-AC (Electro-Technic Products Inc., US), was used to bond the PDMS to a 75 × 50 mm glass slide [15]. The treated surfaces of both substrates were then pressed together and left untouched for at least 1 h at 45 °C for the glass-PDMS surface interface to bond properly. Optimal bond strength was observed when the glass-PDMS cartridge (Fig. 1c) was left overnight. All reagents and glass slides were purchased from Sigma Aldrich unless specifically stated.

### Platform design and fabrication

**Fig. 2:**
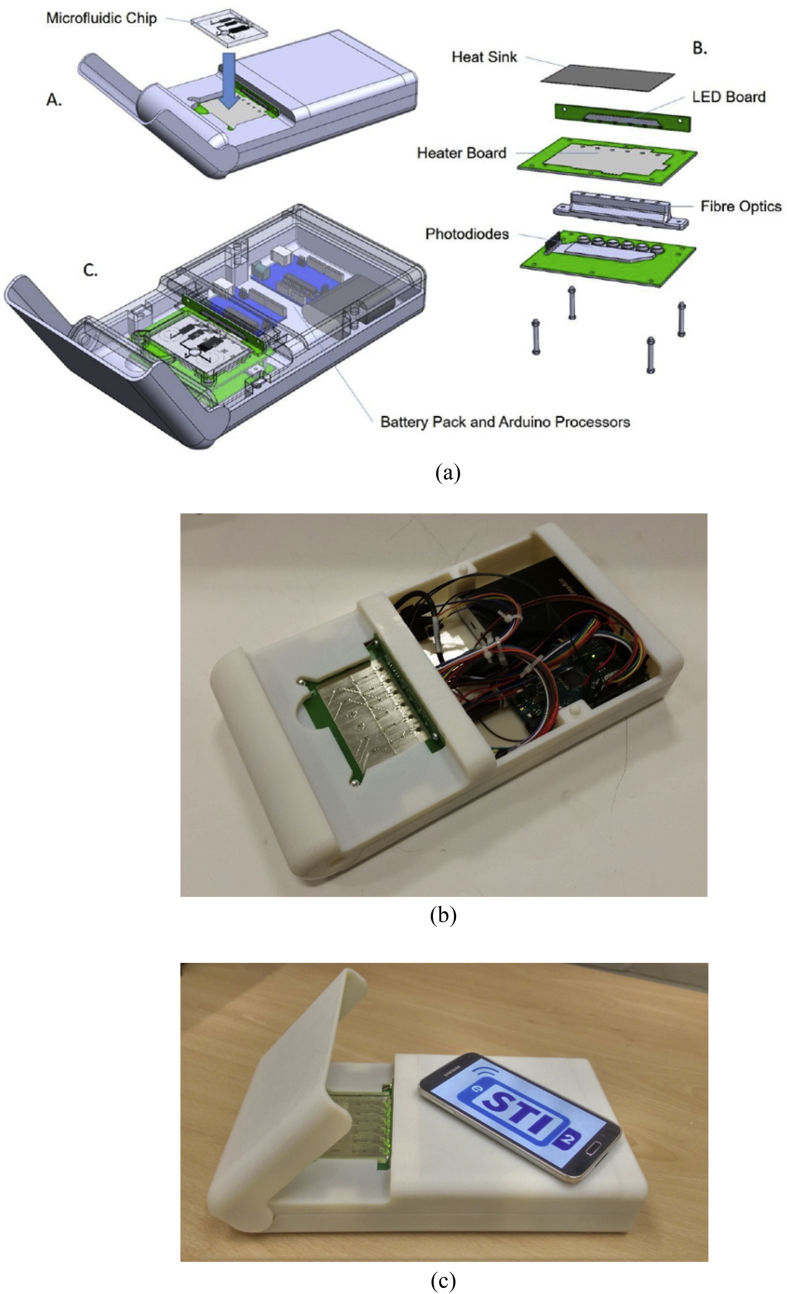
(a) A graphical overview of the POC platform incorporating a USB battery, Arduino for electronic control and data capture, a joule heated stage, LEDs, light pipes and photodiodes; (b) the completed platform showing the isothermal heater and internal components [3,16]. (c) The final device cycling through the RGB LEDs with mobile device (Samsung Galaxy S5) for size comparison.

A shoebox sized device was designed and developed with dimensions of 290 × 162 × 50 mm (Fig. 2a). The device was manufactured using an Objet 30 Pro 3D printer (Statasys, Germany). Three custom circuit boards were designed using Eagle software. The boards incorporate an LED board for excitation of the fluorophore, which is composed of six Avago tri-colour LEDs and a 60 Ω resistor, the blue LED is used for excitation of the fluorophore with a wavelength of 470 nm [16]. The isothermal heater was modified from previous work [3], it used a serpentine track and Joule heating via a high power MOSFET (L2703). Eighteen surface mount resistors (TDK, 10 k ohm 1000 NTC) were placed across the heater; although, only the six adjacent to the amplification chambers were used to measure the temperature in experiments. A bang-bang control programme was run via the Arduino to control the isothermal heater. A 3 mm aluminium plate was placed on top of the isothermal circuit board using a heat transfer adhesive. This ensured good thermal transfer across the entire fluidic device. The third circuit board contains all components for fluorescence detection, all components remain unchanged from Craw et al., this includes the photodiode (BPW21, Centronic, UK), high gain operational amplifier (OPA4750, Texas Instruments, USA) and a 1G ohm feedback resistor [3]. A glass longpass filter (OG515, Schott, Germany) with a wavelength cutoff of 515 nm was placed between the light pipe and the photodiode. A fibre optic housing was 3D printed in VeroBlack to hold six 3 mm diameter light pipes, which transmit the signal from the amplification chamber to the photodiodes. The fibre optic housing and threaded hex spacers brought the main body of the isothermal platform together. All control and data acquisition was implemented via an Arduino Mega. The Arduino Mega was powered by a 10 Ah USB battery using 5 V DC and 1 A current. All electronic components were acquired from RS, UK unless stated.

### DNA extraction and amplification – RPA basic kit

Chitosan membranes were created by dissolving 1 w/v% chitosan in 2 v/v% acetic acid and stirred for 4 h. Whatman chromatography paper was used as a substrate and was cut to shape using a cutting tool and press, and placed in the solution with 1v/v% glutaraldehyde for 8 h. Following cross linking of chitosan to the cellulose membrane, the membranes were removed from solution, rinsed in a 10 mM acetic acid solution to remove any unbound chitosan and dried in an oven at 60 °C for 1 h.

A 2-(N-morpholino)ethanesulfonic acid (MES) buffer (10 mM) was prepared at pH 5.0 for the RPA samples. The positive and negative control DNA samples from the TwistAmp Basic kit (TwistDx, UK) were used in first set of experiments to validate the fluidic design and thermal stability of the platform. The original TwistDx assay was modified to allow for extraction using the chitosan membrane and larger volumes required (Table 1). Whilst each amplification chamber only holds 25 μL, the total sample used for each reaction chamber was 50 μL to account for the extra volume required due to the chamfered microfluidic inlets and outlets of the chambers. Positive and negative samples were run on different fluidic cartridges. The positive/negative sample mixed with the MES buffer (80 μL) was flowed directly into the chitosan chamber via inlet 1 (Fig. 1a) at a flow rate of 5 μL/s using Fusion 100 syringe pump (KR Analytical, UK). Excess fluid flows to the waste chamber, any remaining fluid was pushed into the waste chamber by the use of pressurised air. Inlet 2 was opened at a flow rate of 5 μL/s and the TwistDx rehydration buffer was released, the alkaline pH of the rehydration buffer releases the DNA back into solution. The solution was passed through the passive valve as the waste chamber was full and solidified. Each chamber filled, the lyophilised was dissolved and amplification was performed at 44 °C. Gel electrophoresis was carried out to analyse the outcome of the amplification experiment, a 3% agarose gel stained with 0.5  μl GelRed ran for 1 h 22 min at 75 V. TwistDx positive sample DNA has an amplicon length of 143 base pairs (bp) (±10%). The ultralow gene ruler ladder ranging from 100 to 300 bp was used at both ends of the gel to compare amplified products to a standardised ladder. The gel depicts the combined results for both the positive and negative controlled experiments.

**Table 1:** Comparisons between the original TwistDx chemical and volume compositions [17] with the altered version used for the PDMS-glass cartridge for DNA amplification.

Twistdx Original Version (1 Reaction)	Original Solution Volume	Twistdx Modified Version (1 Reaction)	Modified Solution Volume	Total Solution Volume (8 Reactions)
Tenth dilution of the positive control DNA (in dH_2_O)	10 μl	MES buffer with positive DNA	10 μl	80 μl
Primer A solution	4 μl	Primer A solution	4 μl	32 μl
Primer B solution	4 μl	Primer B solution	4 μl	32 μl
Rehydration buffer	29.5 μl	Rehydration buffer	29.5 μl	236 μl
Magnesium acetate solution	2.5 μl	Magnesium acetate solution	2.5 μl	20 μl

Total volume	50 μl		50 μl	400 μl

### DNA extraction and amplification – *Chlamydia Trachomatis*

An RPA assay using TwistAmp fpg targeting the gene of *CDS2* of CT was designed according to Krõlov et al. [10]. A sequence specific probe for real time optical detection was designed as described in the test manual [18]. The sequences of the primer sets and probe are indicated in Table 2. The RPA reaction mixtures were made as previously described in Section 2.3, however, the samples were now a positive control, negative control, 1 copy/μL CT and 10 copies/μL CT (CT was obtained from Zeptometrix, USA). A slightly alternative protocol was used for this experiment samples as the cells required lysis. The 10 mM MES buffer was prepared at pH 3.0. The cartridge was placed in the POC device, thermal heating initiated at 44 °C and data collection begun.

**Table 2:** *Chlamydia trachomatis* primer and probe sequences used for fpg RPA isothermal amplification assays.

Primer/Probe Name	Size (bp)	Sequence (5’ – 3′)
CDS2-FW (Forward primer)	33	CCT TCA TTA TGT CGG AGT CTG AGC ACC CTA GGC
CDS2-RV (Reverse primer)	32	CTC TCA AGC AGG ACT ACA AGC TGC AAT CCC TT
fpg modified Probe	33	[5′ BHQ1] GTT T [dR-FAM] T ACT CCG TCA CAG CGG TTG CTC GAA GCA [3′-block]

## Results and discussions

**Fig. 3:**
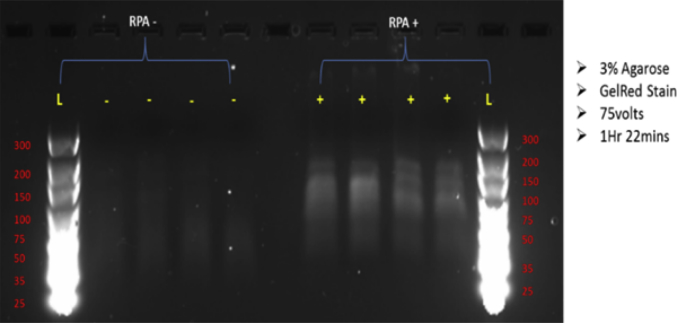
RPA amplicons detected by agarose gel electrophoresis using GelRed. The RPA positive wells contained the positive sample provided with the TwistAmp Basic kit, the negative control wells contained nuclease free water as the sample.

From six chambers run in each experiment a total of four samples were successfully extracted for post amplification analysis from both the RPA positive and negative experiment. Faint bands were shown for positive samples, signifying evidence of DNA amplification within the range of 100–200 bp, therefore in the range of the RPA positive control of 143 bp (Fig. 3). The negative samples show no indication of DNA amplification.

**Fig. 4:**
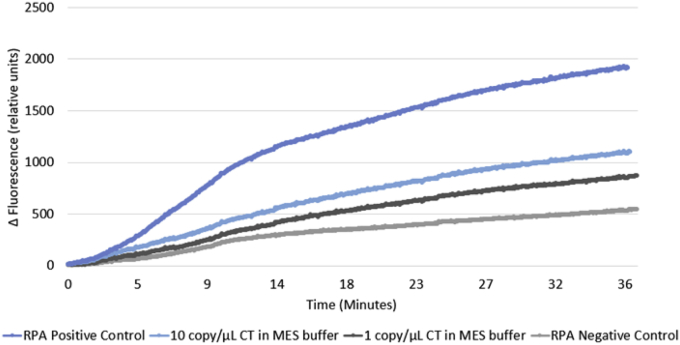
RPA amplification curve of CT (1 copy/μL and 10 copies/μL), RPA positive control and RPA negative control (nuclease free water).

From the CT experiments, it can be seen that there was successful amplification from the positive control along with both 10 and 1 copy/μL of CT in MES buffer (Fig. 4). Amplification initiates within less than 5 min, the positive control shows good amplification after 10 min. For 10 copies/μL of CT optimal amplification occurs after 15 min, the lower copy number, 1 copy/μL takes longer, around 25 min.

Both samples showed successful cell lysis in an acidic buffer, nucleic acid extraction in a continuous, flow through device with waste collected on the cartridge. The nucleic acid was successfully eluted using the amplification rehydration buffer directly from the nucleic acid membrane. Both experiments show the cartridge design to be optimal, the passive valve worked repeatedly and amplification chambers filled, six per run consecutively.

## Conclusion

The work presented in the paper shows a fluidic cartridge that has been modified for sample in to answer out detection using a POC system and an RPA assay. The use of the chitosan membrane and use of the RPA reagents for nucleic acid elution is of particular interest as it simplifies the extraction and amplification process further than previously possible. Results for low copy numbers of CT were gained in 15–25 min. Further work must be conducted using real, complex, samples such as urine or urine with swab samples to ensure results can be replicated.

The hybrid PDMS–glass fluidic cartridge for multiplex DNA amplification reactions showed good capabilities for extraction, waste collection and sample splitting through the multiplexer prior to amplification. The use of a single passive valve avoids the need for any active valves. CFD was used successfully followed by numerous experiments to design the multiplexer with the ability to split the fluid into six equal parts without the need for any external valves.

The use of the Arduino is for prototyping purposes and allows for rapid addition of modular elements and testing including control via a mobile phone device using Bluetooth or visualisation of results on the mobile screen. The platform that has been shown here has been used with other isothermal assays including HDA making this a versatile system. The platform is low cost and battery operated; whilst the device can be held by hand it is still relatively large and in the current form still requires the use of a desktop syringe pump.
